# Activation Effects of Polysaccharides of *Flammulina velutipes* Mycorrhizae on the T Lymphocyte Immune Function

**DOI:** 10.1155/2014/285421

**Published:** 2014-07-14

**Authors:** Zheng-Fei Yan, Nai-Xu Liu, Xin-Xin Mao, Yu Li, Chang-Tian Li

**Affiliations:** ^1^College of Chinese Medicinal Materials, Jilin Agricultural University, Changchun 130118, China; ^2^Engineering Research Center of Edible and Medicinal Fungi, Ministry of Education, Jilin Agricultural University, Changchun 130118, China

## Abstract

*Flammulina velutipes* mycorrhizae have increasingly been produced with increasing of *F. velutipes* production. A mouse model was thus used to examine potential effect of *F. velutipes* mycorrhizae on the immune function. Fifty female Wistar mice (5-weeks-old) weighed 15–20 g were randomly allocated into five groups. Polysaccharide of *F. velutipes* mycorrhizae were treated with mice and mice spleen lymphocytes. The levels of CD3^+^, CD4^+^, and CD8^+^ T lymphocyte, interleukin-2 (IL-2), and tumor necrosis factor-a (TNF-*α*) were determined. The results showed that the proportions of CD3^+^, and CD4^+^ T lymphocyte, the ratio of CD4^+^/CD8^+^, and the levels of IL-2 and TNF-a were significantly increased in polysaccharide of *F. velutipes* mycorrhizae, while the proportion of CD8^+^ T lymphocyte was decreased in polysaccharide of *F. velutipes* mycorrhizae-dose dependent manner. Our findings indicated that a long term exposure of polysaccharide of *F. velutipes* mycorrhizae could activate the T lymphocyte immune function. Polysaccharide of *F. velutipes* mycorrhizae was expected to develop into the immune health products.

## 1. Introduction

*Flammulina velutipes* is one of the most popular edible fungi in China and Japan because of its high nutritional value and attractive taste. It has been reported that both the fruiting bodies and the fungal mycelia of* F. velutipes* contain bioactive polysaccharide with beneficial immunomodulatory, antitumor, and biological activity on hepatocytes [1]. Ko et al. [2] reported that* F. velutipes* contains total carbohydrate (58.0%), protein (27.5%), fat (7.0%), and ash (7.4%). Owing to its high nutritional values and attractive taste, it is one of the most popular edible mushrooms worldwide. Its production and consumption ranked at fourth place in the edible mushrooms in the world [2, 3]. In recent years, its consumption has increased rapidly, and many factories have been established for large-scale artificial cultivating in Asian countries, especially in Japan and China. Most studies focused on the conventional nutrient profiles of* F. velutipes* such as amino acids and vitamins, bioactive macromolecules, and increasing production to obtain the maximum benefit. Few studies were carried out on edible fungi residue and mycorrhizae. Due to the large-scale cultivating, yields of edible fungi residue and mycorrhizae were increased, subsequently. It was a lot of waste. The residue had been reused or recycled again [4–7], but mycorrhizae had no effective utilization. The mycorrhizae had great value space, such as used as organic fertilizer.

In this study, polysaccharide of* F. velutipes* mycorrhizae (PFVM) role in immune regulation was investigated [8]. It could develop new immune health products. Polysaccharide of* F. velutipes* mycorrhizae (PFVM) was obtained using hot water. Preliminary structural characterization of polysaccharide was then conducted via physicochemical property, Fourier transform infrared (FTIR) spectroscopy. Finally, the T lymphocyte immune function of polysaccharide was determined in mice and mice spleen lymphocytes, with examining effects of polysaccharide on T lymphocytes subpopulations (measured as levels of CD3^+^, CD4^+^, and CD8^+^ T lymphocyte and the ratio of CD4^+^/CD8^+^) and cytokines (measured as IL-2 and TNF-*α* levels). The main aim of this study was to investigate the application value of polysaccharide of* F. velutipes *mycorrhizae (PFVM).

## 2. Material and Methods

### 2.1. Preparation of Polysaccharide

*F. velutipes* mycorrhizae were donated by Jiangsu Jiang nan Biological Technology Co., Ltd. A coarse powder (20 mesh) was obtained using a mill (XS-02A traditional Chinese medicine superfine pulverizer, Nanjing, China); a subsample (100 g) was heated with 500 mL deionised water, at reflux, for 3 h. The mixture was cooled to room temperature and filtered. The residue was then refluxed with two additional 100 mL portions of deionised water as described above. The filtrate was dialysed using a Cellu Sep T2 tubular membrane (MWCO: 6,000–8,000, Membrane Filtration Products, Inc., Seguin, TX) for 24 h. The retentate was concentrated to a small volume and then mixed with 3 volumes of 95% ethanol to yield a 70% ethanolic solution. The precipitate thus obtained was lyophilised to obtain 69.84 g polysaccharide of* F. velutipes* mycorrhizae (PFVM) (60 mesh) (total sugar). Polysaccharide powder was stored at 4°C for further uses. According to the Pharmacopoeia of the People's Republic of China, 2010 edition [9], polysaccharide of* Ganoderma lucidum* was selected as positive control group and optimum dose was as follows: 2 g/kg body weight for mice, 150 *μ*g/kg for cells [10–12]. We determined that the low, middle, and high dosages of PFVM were 0.5, 2, 4 g/kg body weight for treatment with mice. Foe treatment with mice spleen lymphocytes, the low, middle, and high dosages of PFVM were 50, 200, 400 *μ*g/kg.

### 2.2. Physicochemical Analysis and Composition for the Polysaccharide

PFVM content was determined by the phenol-sulfuric acid method [13]. Physicochemical properties were determined using the following methods: phenol-sulfuric acid test [13], a-naphthol reaction [14], iodination reaction [15], Fehling's test [16], carbazole reaction [17], FeCl_3_ reaction [18], and Coomassie brilliant blue reaction [19]. The organic functional groups of polysaccharide were identified using an FTIR spectrophotometer (FTIR-8400S, Shimadzu Co., Japan) in the 4000–400 cm^−1^ region via the KBr pressed-disk method.

The gas chromatography (GC) system controlled by the Chemstation software and equipped with a 7653B automatic injector consisted of an Agilent 7890A gas chromatograph (Agilent Technologies, Waldbronn, Germany) coupled to a 5975C VL quadrupole mass detector (MS) and a flame ionisation detector (FID). The composition of sugar composition was analyzed by gas chromatography. The polysaccharides were dissolved in 2 M TFA and hydrolyzed at 121°C for 3 h in a sealed glass tube. The solution was evaporated to dryness and then a mixture of methanol-water was added to give a clear solution, which was evaporated again to dryness. Acetylation was carried out with 10 mg hydroxylamine hydrochloride and 0.5 mL pyridine for 30 min at 90°C. A half mL of acetic anhydride was then added with continuously heating and the alditol acetate derivative was analyzed by using a GC-MS.

### 2.3. Treatment with Mice

Fifty healthy female Wistar mice (5 weeks old) weighed 10–15 g were purchased from Animal breeding center, Jilin University. Forty mice were randomly allocated into control group (CG), low dose group (LG), medium dose group (MG), and high dose group (HG) and then were gavaged with 0, 0.5, 2, and 4 g PFVM per kg body weight twice a day for 60 days, respectively, 2 g polysaccharide of* G. lucidum* per kg for mice as positive control group (PG). Each group had 10 mice. All the mice were acclimatized for 1 week before the gavage treatment. Mice were housed in the Engineering Research Center of Edible and Medicinal Fungi, Jilin Agricultural University. The housing conditions were maintained at temperature of 24 ± 10°C, relative humidity at 55 ± 5%, ventilation frequency at 18 times/h, and a 12 h light/dark cycle. The mice were kept in plastic cages (five mice per cage) with soft chip bedding. The size of all the cages is 470 × 300 × 150 mm, large enough for five mature mice. Throughout the experiment, chips were replaced every 3 days and mice were given the drinking water ad libitum. The health status of mice was daily monitored, and body weight of the mice was recorded every 15 days. The experimental protocol was approved by the Ethics Committee on the Use and Care of Animals, Jilin Agricultural University, China.

### 2.4. Cell Culture and Treatment

Mice spleen lymphocytes were isolated from mouse spleen and cultured according to Mukherjee et al. [20]. The cells culture medium were fed three times a week and subcultured by trypsinisation after beginning to adhere and grow 3 days and then seeded at the appropriate numbers counted with BD Accuri C6 (BD, USA) into wells of cell culture plates for further experiments.

### 2.5. The Weight Ratio of the Thymus, Spleen in Mice

The weight ratio of the thymus and spleen calculated according to the following formula [21]:

(1) weight of an organ  (mg)body weight of a mouse  (mg)×100.

### 2.6. Cell Viability Assay (MTT Assay)

To determine the safety of PFVM, the viability of cells following treatment with different consternations of PFVM was determined by the MTT assay according to Chan et al. [22].

### 2.7. Flow Cytometry

The proportions of CD3^+^, CD4^+^, and CD8^+^ T lymphocyte in the periphery blood, spleen, and thymus in mice and mice spleen lymphocytes were detected by flow cytometry according to Caraher and Giraldo et al. [23, 24]. The ratio of CD4^+^/CD8^+^ T lymphocyte was then calculated.

### 2.8. Cytokine Dosages

A sample of 1 mL of blood of mice was used to obtain serum. Levels of IL-2 and TNF-*α* were detected using 125I Radioimmunoassay (RIA) kits (New Bay Biological Technology Co., Ltd., Tianjin, China).

### 2.9. Statistical Analysis

Statistical analyses were done using SPSS 18.0 package programmer (SPSS Inc., Chicago, IL, USA). The *P* values of less than 0.05 were considered significant, *P* values of less than 0.01 were considered markedly significant, and *P* values less than 0.005 were considered highly significant.

## 3. Result

### 3.1. Polysaccharide Content, Physicochemical Property, and FTIR Analysis

Table 1 showed the main components and physicochemical property of PFVM. The results from the phenol-sulfuric acid test, a-naphthol reaction, iodination reaction, Fehling's test, carbazole reaction, FeCl_3_ reaction, and Coomassie brilliant blue test were presented. Polysaccharide, protein, and reducing sugar existed in the PFVM while no phenol hydroxyl, starch, uronic acid, and enolic hydroxyl existed in it. Polysaccharide content was determined by the phenol-sulfuric acid method; yield of polysaccharide was 23.41% in total sugar and the content of protein of PFVM was determined as 1.53%. Analysis of sugar composition was performed by GC-MS; it indicated that they were composed of L-fucose, D-mannose, D-glucose, and D-galactose in molar ratios of 1.4 : 1 : 1.3 : 1.7. FTIR spectroscopy was typically used for the qualitative measurement of organic functional groups, especially OH, NH, and CO. There were stretching vibrations of OH and saturated CH at 3300–3500 and 2927–2930 cm^−1^, respectively. Two amide bands, one at around 1640 cm^−1^ for amide I (for CO) indicating that the PFVM had conjugated proteins. The group of bands extending from 1485 cm^−1^ to 1350 cm^−1^ was assigned to CH(OCH_2_) flexural vibrations. The absorption band at 1000–1200 cm^−1^ suggested that PFVM contained pyranose monomers in their structures. The bands in the range of 350–600 cm^−1^ were assigned to skeletal modes of pyranose rings [25].

### 3.2. The Effects of PFVM on Changes of Body Weight and the Weight Ratio of the Thymus and Spleen

**Figure 1:**
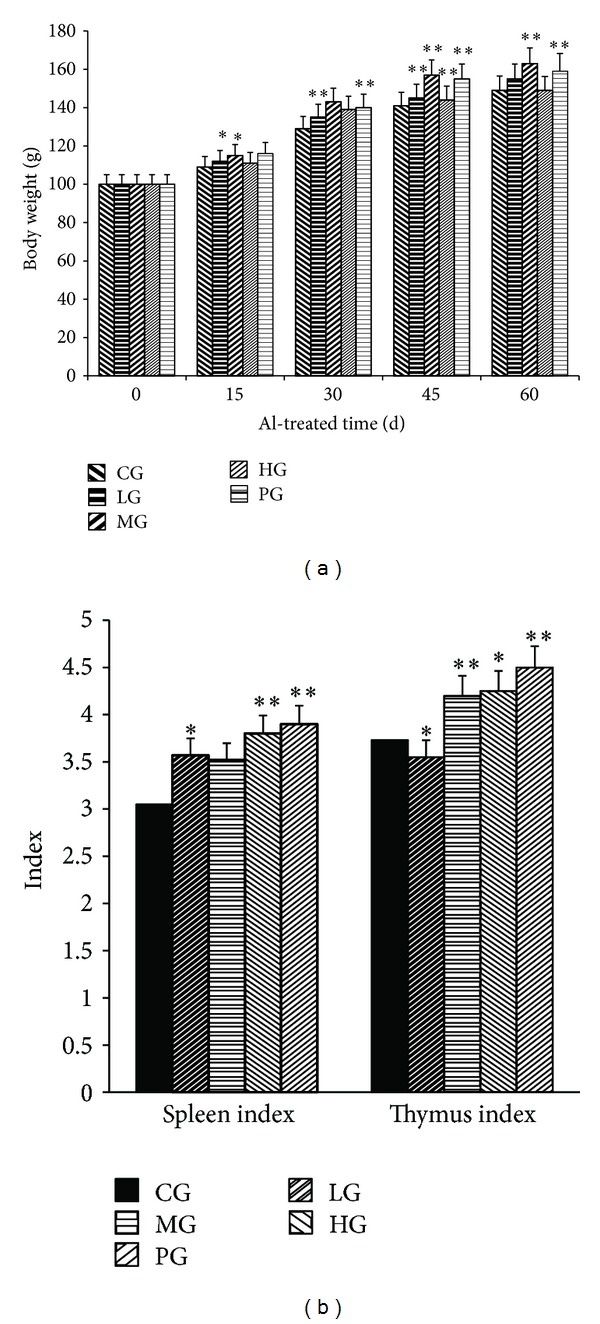
Effect of PFVM on changes of body weight (a) (body weight before treatment with PFVM was set at 100%) thymus index and spleen index (b) in mice. CG, control group; LG, low dose group; MG, medium dose group; HG, high dose group; PG, positive control group. *P < 0.05, **P < 0.01 versus control group.

The body weights of mice in five groups increased continuously throughout the experimental period, and the body weights of PFVM-treated mice were significantly larger than those in CG (Figure 1(a)). As reported in Figure 1(b), the weight ratio of the thymus and spleen in mice treated with PFVM at the doses of 0.5, 2, and 4 g/kg is presented. The weight ratio of the spleen was significantly increased in the mice at the dose of 4 g/kg, and the weight ratio of the thymus for 2 g/kg.

### 3.3. The Effects of PFVM on Thymocyte and Splenocyte Cell Subpopulations and Levels of IL-2 and TNF-*α*

The test conducted on mice confirmed the modulating effect of PFVM on T cells in the central lymphatic organ and peripheral lymphatic organs. The results of the study showed that there was a relationship between the effect induced by PFVM and the dose applied.

**Figure 2:**
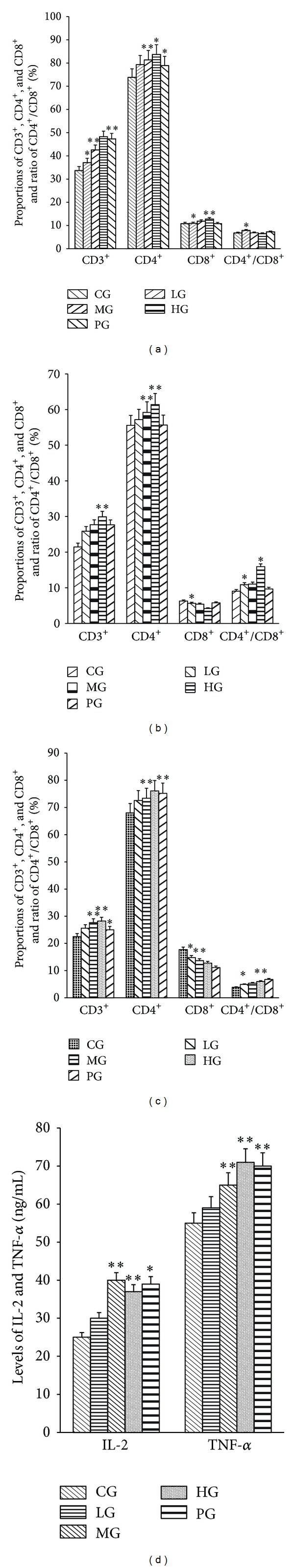
Effects of PFVM on T lymphocyte subpopulation in peripheral blood (a), thymus (b), spleen (c), and levels of IL-2 and TNF-*α* in the serum of mice (d). CG, control group; LG, low dose group; MG, medium dose group; HG, high dose group; PG, positive control group. *P < 0.05, **P < 0.01 versus control group.

T-cell subpopulations after administration of PFVM in peripheral blood of mice were shown in Figure 2(a). The percentage of the CD3^+^ and CD4^+^ was increased in PFVM-dose dependent manner. There was little variation in the percentage of the CD8^+^ and CD4^+^/CD8^+^. The percentage of the CD3^+^ and CD4^+^ was significantly increased with 42.56, 81.33% at MG. Effect of PG on T cell subpopulations was similar to experiment group. At the same time, the effects of PFVM on T cells in thymus were shown in Figure 2(b). The percentage of the CD3^+^, CD4^+^, and CD4^+^/CD8^+^ was increased and the percentage of the CD8^+^ was decreased in PFVM-dose dependent manner. PFVM significantly decreased the percentage of the CD8^+^ with 29.92% at LG, the percentage of the CD3^+^ and CD4^+^ significantly increased at HG with 29.92% and 61.40%. The percentage of the immature the CD4^+^ was significantly increased at MG with 64.30%.  Figure 2(c) showed that the percentage of the CD3^+^, CD4^+^, and CD4^+^/CD8^+^ was increased and the percentage of the CD8^+^ was decreased in PFVM-dose dependent manner in spleen of mice. The percentage of the CD3^+^ and CD4^+^ significantly increased at MG with 28.42% and 73.40%. PFVM significantly decreased the percentage of the CD8^+^ with 29.92% at MG. PG showed a dose-response effect similar to PFVM.

Levels of IL-2 and TNF-*α* in the serum of mice increased in PFVM-dose dependent manner (Figure 2(d)). And levels of IL-2 and TNF-*α* were significantly higher in MG and HG than CG. Level of IL-2 in MG was higher than that in HG, but level of TNF-*α* in HG was higher than that in MG.

### 3.4. The Effects of PFVM on Cell Viability in Spleen Lymphocytes

**Figure 3:**
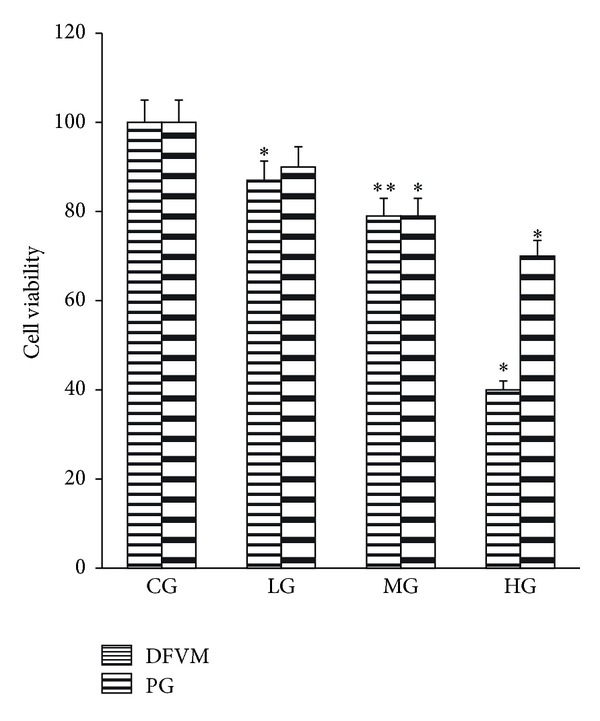
Effect of PFVM on cell viability. (CG was set at 100%). CG, control group; LG, low dose group; MG, medium dose group; HG, high dose group; PG, positive control group. *P < 0.05, **P < 0.01 versus control group.

The effects of PFVM on mice spleen lymphocytes viability was shown in Figure 3. The results showed that, in the cell viability (MTT) assay, LG did not have any appreciable cytotoxic activity. Because HG had the greatest cytotoxic effect, so HG was not investigated further. PG showed a dose-response effect similar to PFVM.

### 3.5. The Effects of PFVM on Splenocyte Cell Subpopulations and Levels of IL-2 and TNF-*α* in Spleen Lymphocytes

**Figure 4:**
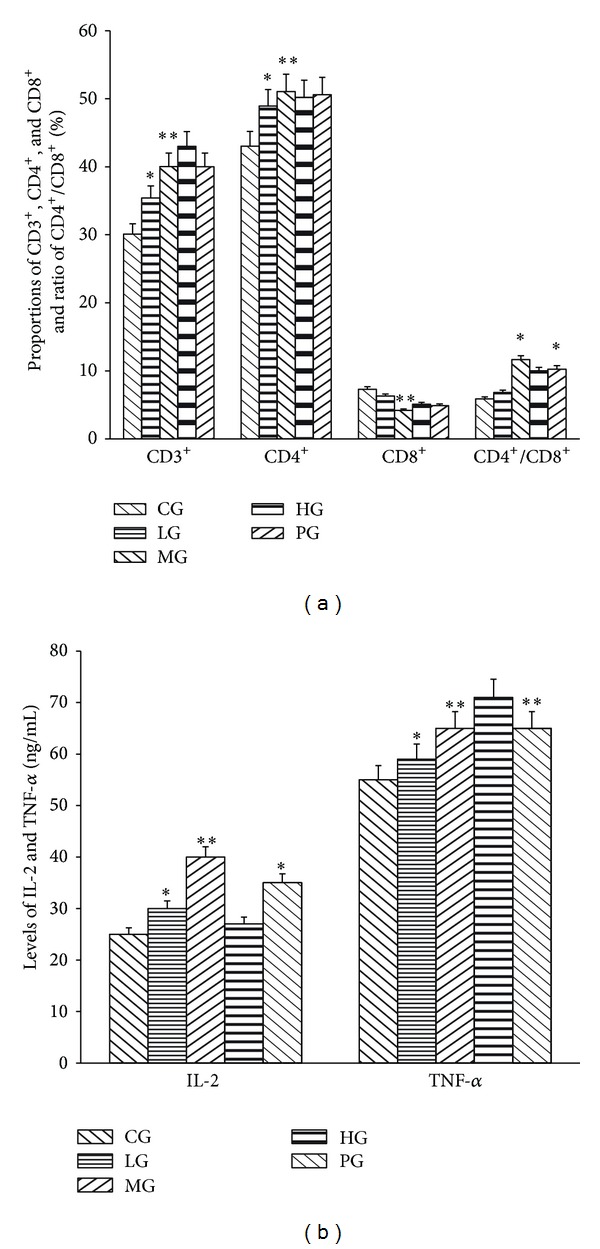
Effects of PFVM on T lymphocyte subpopulation (a), levels of IL-2 and TNF-*α* (b) in mice spleen lymphocytes. CG, control group; LG, low dose group; MG, medium dose group; HG, high dose group; PG, positive control group. *P < 0.05, **P < 0.01 versus control group.

Figure 4(a) showed the percentage of the CD3^+^, CD4^+^, and CD4^+^/CD8^+^ was increased and the percentage of the CD8^+^ was decreased in PFVM-dose dependent manner. The percentage of the CD3^+^ and CD4^+^ was significantly increased with 39.4% and 52.1% on MG. However, the strongest decreasing effect of PFVM on CD8^+^ was observed with 4.23% when PFVM was administered at the dose of MG.

Levels of IL-2 and TNF-*α* increased in PFVM-dose dependent manner in PFVM-treated cells. And levels of IL-2 was highest in MG. Level of TNF-*α* was highest in HG (Figure 4(b)).

## 4. Discussion

In our research, PFVM could promote the immune function of erythrocytes in the blood of mice. T lymphocyte immune function was also affected by PFVM. And this experiment highlighted the relationship between PFVM and T lymphocyte immune function, aiming to increase awareness of a possible advantage of exposure to PFVM. There was no death in the PFVM-treated mice during the experimental period. However, PFVM-treated mice were more active than those in CG. The increasing in the body weight again indicated that PFVM could promote the growth of mice.

T lymphocytes stayed and matured in the thymus and spleen [26] and PFVM could enhance the structure and function of these organs.

T lymphocyte subpopulation played an important role in the T lymphocyte immune function [27, 28]. The CD3^+^ was a marker for the T lymphocytes, and the proportion of the CD3^+^ T lymphocyte could reflect the number of T lymphocytes. T lymphocyte subpopulation could be divided into two groups: T helper lymphocyte and T inhibiter lymphocyte. CD4^+^ was a marker for T helper lymphocyte. The CD4^+^ T lymphocyte could activate T lymphocytes and induced cellular immune responses. CD8^+^ was a marker of T inhibiter lymphocyte. The CD8^+^ T lymphocyte had cytotoxic effect and could suppress the activity of T lymphocytes. The ratio of CD4^+^/CD8^+^ was thus reflecting activate/inhibitory functions of the immune system of mice [29]. In this experiment, the proportion of the CD3^+^ and CD4^+^ T lymphocyte increased. Meanwhile, the CD8^+^ T lymphocyte decreased in the PFVM-treated mice or cells. So our results indicated that PFVM could promote T lymphocyte immune function partially by increasing the T cell subpopulation.

IL-2 was an important cytokine involved in the growth of T lymphocytes. In this experiment the level of IL-2 was increased by PFVM. IL-2 and TNF-*α* were secreted by T help-1 cell subpopulation. TNF-*α* was also secreted by macrophages. Both cytokines could modulate the T lymphocyte immune function [30]. PFVM increased the production of IL-2 and TNF-*α*. Studies on both mice and cells indicated that PFVM promoted the levels of IL-2 and TNF-*α*. Levels of IL-2 and TNF-*α* was increased in PFVM-dose dependent manner. IL-2 and TNF-*α* reacted with each other; if one increased, the other should increase. We found that the proportion of CD4^+^ increased in this experiment. This ascend may contribute to the increasing of IL-2 and TNF-*α*.

## 5. Conclusion

The increases of the proportion of the CD3^+^ and CD4^+^, ratio of CD4^+^/CD8^+^, and levels IL-2 and TNF-*α* and the decrease of the CD8^+^ T lymphocyte indicated that PFVM could promote the T lymphocyte immune function.

## Figures and Tables

**Table 1:** Comparison of the yields and basic physicochemical characteristics of PFVM (+ positive, − negative).

	PFVM
Polysaccharide content (%)	23.41
Protein content (%)	1.53
Sugar components (mol%)	
L-Fucose	23.41
D-Mannose	15.64
D-Glucose	20.43
D-Galactose	25.08
Phenol-sulfuric acid test	+
a-Naphthol reaction	−
Lodination reaction	−
Fehling's test	+
Carbazole reaction	−
FeCl_3_ reaction	−
Coomassie brilliant blue reaction	+
